# Optimal Connection for Tiotropium SMI Delivery through Mechanical Ventilation: An In Vitro Study

**DOI:** 10.3390/pharmaceutics12030291

**Published:** 2020-03-24

**Authors:** Tien-Pei Fang, Yu-Ju Chen, Tsung-Ming Yang, Szu-Hu Wang, Ming-Szu Hung, Shu-Hua Chiu, Hsin-Hsien Li, James B. Fink, Hui-Ling Lin

**Affiliations:** 1 Department of Respiratory Therapy, Chang Gung Memorial Hospital, Chiayi 61301, Taiwan; pig61210@cgmh.org.tw (T.-P.F.); ahui0313@gmail.com (S.-H.W.); chiu@cgmh.org.tw (S.-H.C.); 2 Department of Respiratory Care, Chang Gung University of Science and Technology, Chiayi Campus, Chiayi 61301, Taiwan; 3 Department of Pediatrics, National Cheng Kung University Hospital, Tainan 70402, Taiwan; s18908005@gmail.com; 4 Department of Pulmonary and Critical Care Medicine, Chang Gung Memorial Hospital, Chiayi 61301, Taiwan; n120633@cgmh.org.tw (T.-M.Y.); m12049@cgmh.org.tw (M.-S.H.); 5 Department of Respiratory Therapy, Chang Gung University, Taoyuan 33302, Taiwan; hsinhsien@mail.cgu.edu.tw; 6 Aerogen Pharma Corp., San Mateo, CA 94402, USA; fink.jim@gmail.com

**Keywords:** Soft mist inhaler, mechanical ventilation, adapter, inhaled dose, in vitro

## Abstract

We aimed to quantify Soft Mist Inhalers (SMI) delivery to spontaneous breathing model and compare with different adapters via endotracheal tube during mechanical ventilation or by manual resuscitation. A tiotropium SMI was used with a commercial in-line adapter and a T-adapter placed between the Y-adapter and the inspiratory limb of the ventilator circuit during mechanical ventilation. The SMI was actuated at the beginning of inspiration and expiration. In separate experiments, a manual resuscitator with T-adapter was attached to endotracheal tube, collecting filter, and a passive test lung. Drug was eluted from collecting filters with salt-based solvent and analyzed using high-performance liquid chromatography. Results showed the percent of SMI label dose inhaled was 3-fold higher with the commercial in-line adapter with actuation during expiration than when synchronized with inspiration. SMI with T-adapter delivery via ventilator was similar to inhalation (1.20%) or exhalation (1.02%), and both had lower delivery dose than with manual resuscitator (2.80%; *p* = 0.01). The inhaled dose via endotracheal tube was much lower than inhaled dose with spontaneous breathing (22.08%). In conclusion, the inhaled dose with the commercial adapter was higher with SMI actuated during expiration, but still far less than reported spontaneous inhaled dose.

## 1. Introduction

Aerosol therapy plays a critical role in treating pulmonary and non-pulmonary diseases. Currently, more than 250 inhalers are available for delivering drugs as a component of treatment [1,2]. The soft mist inhaler (SMI) is a propellant-free, mechanically operated, multiple-dose device, which nebulizes a liquid drug solution through a uniblock, which allows the drug solution to produce aerosol in a slow-moving cloud with a spray time of 1.0–1.5 s [3]. The low aerosol velocity reduces oropharyngeal deposition and offers the benefits of generating more than 60% of the fine-particle fraction (≤ 5.0 µm) independently of inspiratory effort for the delivered bronchodilator [3,4].

Tiotropium bromide SMI, a long-acting muscarinic agent (LAMA) once-a-day treatment, indicated as a maintenance bronchodilator to relieve the symptoms of chronic obstructive pulmonary disease (COPD) [5]. SMIs are designed for spontaneous breathing patients. However, no LAMA is available for intubated COPD patients. LAMAs are often prescribed for continuation of the COPD regimens for intubated patients. Yet, many barriers, such as the required interface and contour of the ventilator circuit and endotracheal tube (ETT), hinder aerosol delivery to a ventilator-supported patient [6]. Dellweg evaluated SMI in vitro to compare drug delivery using an SMI prototype adapter, modified connectors, and holding chamber designed for metered-dose inhaler (MDI), using flow profiles of mechanically ventilated COPD patients. Their results showed that the prototype in-line adapter delivered a greater drug dose than the other accessory device combinations in fine particle deposition; however, this prototype adapter is not commercially available [7].

Inhaled drug dose efficiency and optimal connection of an SMI to a ventilator system remain unknown. Clinicians try to connect the SMI to the ventilator circuit in various creative ways. Currently, an in-line adapter for the SMI is commercially available to provide an access port in the ventilator circuit that facilitates the administration of medication from the SMI as prescribed to mechanically ventilated adult patients [8]. The efficiency of administering SMI with this in-line adapter has not been reported. We hypothesized that, with the long aerosol release time of the SMI, the release of aerosol emitted from the SMI during expiration of the ventilator system may increase the delivered dose compared release during inspiration. Therefore, we aimed to quantify SMI delivery to spontaneous breathing model and compare with different adapters via endotracheal tube with a range of actuation timing during mechanical ventilation or by manual resuscitation.

## 2. Materials and Methods

### 2.1. Lung Model

A Dräeger V300 ventilator (Dräeger Medical Systems, Lübeck, Germany) operated with volume control, tidal volume of 500 mL, respiratory rate of 15 breaths/min, inspiratory time of 1.3 s, inspiratory flow of 50 L/min in square flow waveform, positive end-expiratory pressure of 5 cmH_2_O, and inspired oxygen fraction of 0.21. A heated humidifier (Fisher and Paykel Healthcare, Auckland, New Zealand) was set to invasive auto-mode with a temperature of approximately 37 °C. The ventilator circuit was connected to a 7.5-mm ETT, attached to a collecting filter connected to a passive test lung model (Michigan Instruments Inc., Kentwood, MI, USA) with resistance of 5 cmH_2_O/L/min and compliance of 50 mL/cmH_2_O, simulating an adult COPD patient.

### 2.2. SMI Delivery in the Ventilator System

**Figure 1:**
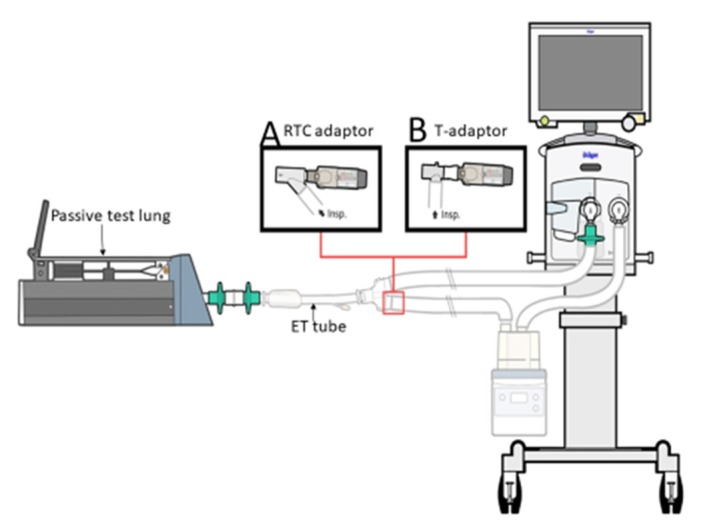
Experimental set-up. A ventilator was connected heated humidified adult circuit to an endotracheal tube attached to a collecting filter and passive test lung. The soft moisture inhaler was placed between the inspiratory limb and Y-adapter with an in-line adapter (**A**) and a T-adapter (**B**).

**Figure 2:**
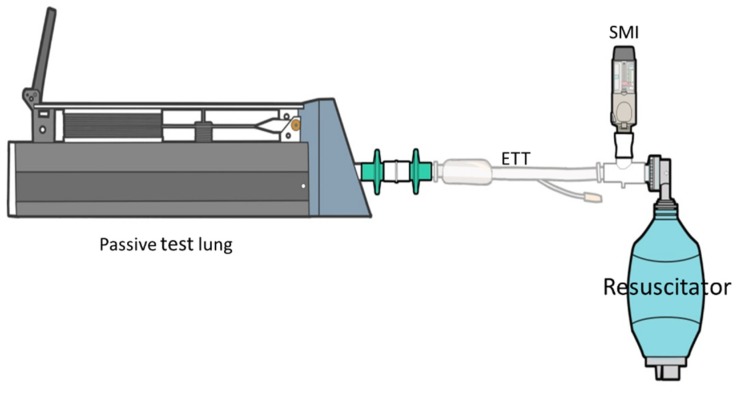
A soft moisture inhaler delivered through a T-adapter between the endotracheal tube and a manual resuscitator.

The SMI, containing tiotropium with a label claim of 2.5 μg/actuation (Boehringer-Ingelheim, Ingelheim, Germany) was connected as follows: (1) A commercial in-line adapter (Instrumentation Industries Inc., Bethel Park, PA, USA) was placed between the Y-adapter of the ventilator circuit, and the inspiratory circuit was at an angle of 45° from the Y-adapter (Figure 1A). The SMI was placed at the medication inlet port, which was designed in an oval shape to fit the SMI, and then parallel to the Y-piece inspiratory limb [8]. (2). A T-adapter commonly used for a jet nebulizer was placed between the Y-piece of the ventilator circuit and the inspiratory limb (Figure 1B). The SMI was connected to the T-adapter by a silicon adapter; thus, the SMI was parallel to the Y-piece and 90° from the inspiratory limb. (3) T-adapter, used for a jet nebulizer, was placed between the ETT and a manual resuscitator to deliver the drug directly to the airway (Figure 2). The resuscitator was manually squeezed with a pressure of 20 cmH_2_O, synchronized with each inspiration, based on the ventilator waveform.

Each SMI was primed with 3 actuations prior to testing. Four actuations (puffs) at 30-s intervals were administered, synchronized with the beginning of inspiration and with beginning of expiration from the ventilator. With the manual resuscitator, two puffs SMI actuated at beginning of inspiration. Each experiment was repeated for five times (*n* = 5).

**Figure 3:**
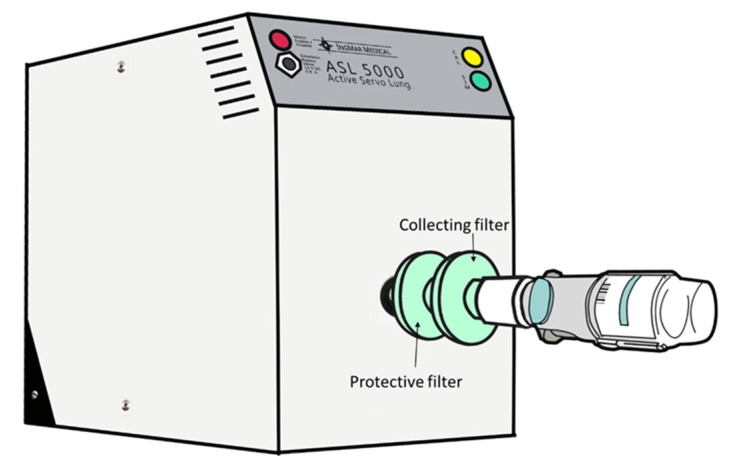
The experimental setup of spontaneous breathing model. A soft moisture inhaler attached to a collecting filter and then the breathing simulator.

To quantify inhaled dose of SMI with spontaneous breathing of COPD patients without an artificial airway, a model of a breath simulator (ASL 5000; IngMar Medical Inc., Pittsburgh, PA, USA) was set to adult parameters of 500 mL tidal volume and respiratory rate of 15 breaths/min with resistance of 0.8 cmH_2_O/L/min and compliance of 53 mL/cmH_2_O, and an inspiration phase 1.3 s with a sinusoidal waveform (Figure 3). A collecting filter was placed between the SMI and the inlet of the simulator. The SMI was actuated twice, with 30-s intervals, at beginning of inspiration.

### 2.3. Drug Elution and Analysis of Tiotropium

To calculate the extraction rate of the collecting filter, 1 actuation (2.5 ug) of tiotropium was delivered to the filter and eluted using 10 mL of an acid sodium salt solvent [9]. The drug dose was analyzed using high-performance liquid chromatography (HPLC), and the extraction rate of the filter was 95%. The drug trapped by the collecting filter was eluted with 10 mL of solvent and gently agitated for 1 min.

Tiotropium was analyzed using a validated HPLC method and an Alliance e2695 (Waters Corp., Milford, MA, USA) equipped with an ultraviolet detector, auto-sampler, degassing unit, and column oven. The analysis was performed using a C8-3 column (GL Sciences Inc., Torrance, CA, USA) with a particle size of 5 µm. The column oven was set at 25 °C. A 1-heptanesulfonic acid sodium saltwater solution (Sol A) and acetonitrile (Sol B) mixture were used in the mobile phase of the gradient elution. Tiotropium was analyzed at a detector wavelength of 240 nm. The injection volume was 100 μL and a flow rate of 2.0 mL/min was used. The tiotropium retention time was 8 min. Eight solutions of varying concentrations were prepared using the United States Pharmacopeia reference standard of tiotropium bromide monohydrate (Sigma-Aldrich Corp., St. Louis, MO, USA). The linearity ranged from 0.0155 to 0.8271 μg/mL (*R*^2^ = 0.9999). The percentage of tiotropium recovered from the working standard solution was 100% at 0.25 μg/mL with a confidence interval of 0.95.

### 2.4. Ventilator Parameter Changes

Air leak from the ventilator circuit with SMI actuation was observed and reported. The leak volume was calculated by subtracting the expiratory tidal volume from inspiratory tidal volume.

### 2.5. Statistical Analysis

The delivered inhaled drug dose was calculated as a percentage of the total label emitted dose per actuation (10 μg). Data were analyzed using the Statistical Package for the Social Sciences version 23.5 (IBM Inc., Armonk, NY, USA), and expressed as means ± standard deviation. Drug doses were compared using a one-way analysis of variance with a Scheffe post hoc test. To understand the impact of the influence of actuation timing, the delivered drug dose of the in-line adapter and T-adapter synchronized with inspiration or expiration were analyzed using an independent t-test. A P-value of 0.05 was considered statistically significant.

## 3. Results

**Figure 4:**
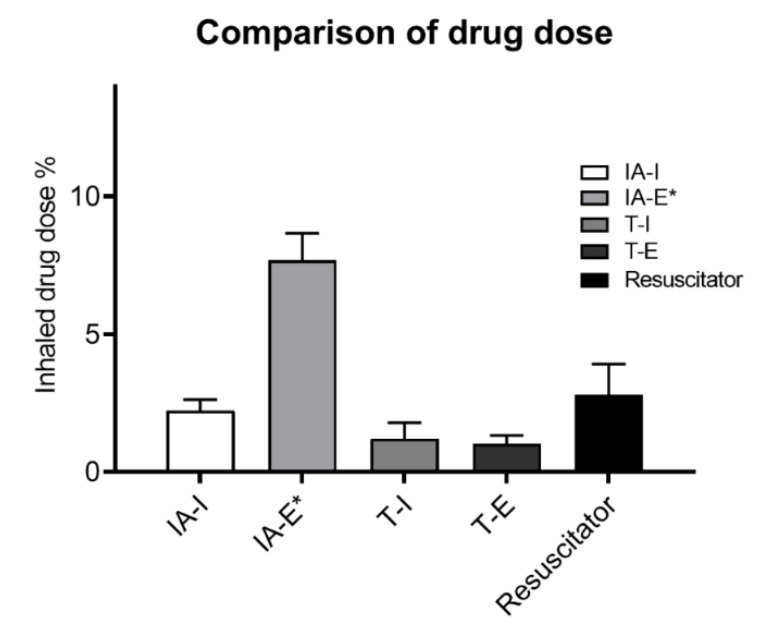
Comparison of the drug dose (%) collected distal to the endotracheal tube using different connections, actuation sequence, and methods of ventilation, * Significantly greater inhaled dose than other connections according to an analysis of variance (*p* < 0.001) and greater dose during expiration than inspiration with the in-line adapter. Abbreviations: IA-I: in-line adapter at inspiration; IA-E: in-line adapter at expiration; T-I: T-adapter at inspiration; T-E: T-adapter at expiration.

The inhaled percent of emitted dose delivered distal to the ETT is shown in Figure 4. With the in-line adapter the inhaled dose (mean ± SD) was greater with actuation during expiration (7.68 ± 0.98%) than synchronized with inspiration (2.22 ± 0.4%; *p* < 0.001). In contrast, the T-adapter inhaled dose via ventilator was similar whether actuation was at the start of inhalation (1.20 ± 0.58%), or at the beginning of exhalation (1.02 ± 0.30%; *p* = 0.557). The drug dose with T-adapter was lower during mechanical ventilation than with manual resuscitator (2.80 ± 1.11%; *p* = 0.01). The inhaled tiotropium dose with simulated spontaneous breathing (22.08 ± 4.8%) was nearly 3-fold greater than administration through the endotracheal tube (*p* < 0.001).

The air-leak volumes of the in-line adapter during actuated were 17.1 ± 5.5 mL (3.4 ± 0.01%) at inspiration and 19.4 ± 2.5 mL (3.8 ± 0.001%) at expiration; and those with T-adapter were 270.6 ± 5.0 mL (54.1 ± 0.01%) at inspiration and 288.7 ± 5.2 mL (57.7 ± 0.01%) at expiration. There was significant difference between two adapters (*p* < 0.001), but no difference between two actuation timing with each adapter (*p* > 0.05).

## 4. Discussion

The present study, the first study evaluated commercial adapter for mechanical ventilation, demonstrated that tiotropium administered with an SMI during mechanical ventilation is most efficiently delivered with the in-line aerosol adapter. SMI with in-line adapter actuated during expiration yielded greater inhaled dose than with the T-adapter. However, the highest inhaled dose during mechanical ventilation was 3-fold less than with our model of spontaneous breathing, and 8-fold less than delivered with a resuscitator.

### 4.1. Influence of Adapter Selection

The SMI was developed and approved for patients with spontaneous breathing. To extend use of the medications for disease management for intubated COPD patients, clinicians have attempted to connect SMI to a ventilator system in various ways before a commercial in-line has been available. However, the drug dose of SMI delivery to a ventilator system with the available system has not been quantified.

SMI adapter designed with separation boards or valves reduces the impact of ventilator flow thus increases drug delivery distal to the ETT. Dellweg et al., using an SMI prototype connector was designed in a 90-degree angle and two flow separation boards that detoured ventilator flow into the aerosol stream, possibly reducing the impact of ventilator flow on the emitted aerosol cloud. The SMI fine particle delivered through connectors and spacers was only 5–15% [7]. Suggett and Nagel assessed a prototype SMI adapter for a ventilator circuit use (RespiConnect™ adapter) with an SMI delivering a combination of salbutamol and ipratropium [10]. The prototype adapter contained a self-sealing valve and safety cap intended to prevent the loss of airway pressure and maintain a closed ventilator circuit. The study reported the recovery of approximately 30% of total emitted drug dose distal to the ETT. However, the prototype adapters from these two studies were not commercially available.

In our study, the maximum inhaled drug dose collected during mechanical ventilation was 7.68 ± 0.98%. The large difference in the delivered dose in our study than Suggett’s study is likely due to the design of the adapters. A secondary consideration may be the shorter inspiration time we set on the ventilator; Suggett set an inspiration time of 1.5 s, compared with it at 1.3 s in the present study. We speculated that part of the aerosol was lost after the ventilator turned to expiration. The SMI connected to the modified T-adapter yielded a lower delivered drug dose during mechanical ventilation than connect to the in-line adapter or manual resuscitator, regardless of the actuation timing. A valved adapter may be associated with less gas ventilation system leakage. During the experiments, we have observed greater volume loss with a T-adapter with obvious gas leakage felt around the SMI during each actuation. The average gas leak of 279.6 ± 10.7 mL with T-adapter is clearly unacceptable for clinical use with mechanically ventilated patients. In contrast, 18.3 ± 4.2 mL leakage with the in-line adapter may be considered tolerable, but is less than optimal. Using a T-adapter with an SMI during mechanical ventilation compromises the operation of the ventilator and could place patient in unacceptable risk. Thus, a T-adapter as used in this study is not recommended for delivery SMI during mechanical ventilation.

### 4.2. Influence of Actuation Timing

The effect of poor synchronization is based on MDI devices emitting large particles with high initial velocities. Previous studies recommend that actuation of an MDI should be synchronized with the precise beginning of inspiration to improve drug delivery [11,12]. Mechanical ventilators are commonly designed with a bias flow to allow patient trigger with less lag time and work of breathing. The interaction of SMI actuation synchronization and bias flow during mechanical ventilation affects aerosol deposition. Ke reported greater delivered dose during mechanical ventilation with SMI actuation at the beginning of expiration rather than at beginning of inspiration (16.1% vs. 9.8%, retrospectively) [13]. Similarly, we found a significant increase in the aerosol delivery of SMI with the in-line adapter with actuation at the beginning of expiration. The SMI aerosol spray exits the nozzle for a duration of 1.5 s [14]. In our study, the respiratory cycle was set to 4 s and 1.3 s inspiration time. With the bias flow at 2.0 L/min, gas travels 33.3 mL/s and a total of 90 mL for 2.7 s expiratory time. The measured distance and volume from SMI outlet to the collecting filter was 93 mL, including the in-line adapter, Y-adapter, and the ETT. When the SMI was actuated synchronized at expiration, the aerosol had sufficient time to be released into the ventilator circuit while airway pressure was low and leak of gas through the adapter was minimal. The aerosol could be pushed forward to the ETT during expiratory phase, resulting in a significantly greater drug dose. In contrast, the inspiration time in the present study was shorter than the release time of the SMI reducing the proportion of aerosol passing through the ETT before end of inspiration, with aerosol pushed from the airway with transition to expiration.

### 4.3. Manual Resuscitation Bag Delivery Technique

Manual resuscitators can be used for aerosol delivery to achieve spontaneous breathing in tracheostomized children [15,16]. This method of drug administration could be employed when doses of bronchodilators need to be rapidly administered to a patient with an artificial airway [17]. Ari reported that, in both tracheostomy and endotracheal tube spontaneous breathing models, manual resuscitation bag provides ventilation with prolonged inflation breaths producing 3-fold greater albuterol delivery dose than through T-adapter or Tr.-collar [18]. In contrast, SMI delivery via a manual resuscitator yielded the second highest drug dose among all of the methods compared, but still 8 fold less than spontaneous breathing. The aerosol was expected to be delivered directly to the ETT by a manual resuscitator; however, without a valved adapter, gas leakage was observed around the SMI during actuation, yielding a relatively small delivered drug dose (2.80 ± 1.11%). Additionally, two operators were required to administer the aerosol treatment, one with the resuscitator squeezing and one for hold the ETT and actuating the SMI. Therefore, administration of SMI with resuscitator is not recommended.

### 4.4. Clinical Implication

A shorter inspiration time, smaller tidal volume, and possible slower inspiratory flow may reduce SMI delivery during mechanical ventilation. Newman et al. first reported lung deposition of SMI using gamma scintigraphy among healthy nonsmoking subjects [19]. They reported a median dose of 39.2% of the whole lung deposition, ranged between 20–60%, with a mean inspiratory time of 6.7 s and a mean tidal volume of 2.6 L. Our study showed only 22.8% with spontaneous tidal breath model with 1.3 s duration of inhalation, yet the sinusoidal waveform generated negative flow at the first two-third of inspiration before a phase.

The relatively low inhaled dose of aerosol from the SMI in our study raises concern of whether sufficient drug delivery can be achieved to justify its use during mechanical ventilation. In our model, only a fraction of the spontaneous breathing dose was achieved during mechanical ventilation. Based on our findings, the commercial adapter was marginally effective and would require multiple doses to achieve target lung dose. While the commercial product yields greater drug delivery whereas the handmade connector (T-adapter), the drug delivered distal to the ET is far less than expected with spontaneously breathing patients. If the SMI with commercial adapter is used, our finding suggested that the SMI should be actuated at beginning of expiration for greater drug delivery efficiency. While drug delivery with a manual resuscitator could be substituted, with slow breath and larger tidal volume incorporated with SMI release time, the achieved dose is so low that one might consider using an alternative aerosol delivery strategy.

### 4.5. Limitations

This study was not without limitations. The manufacturer produces SMIs with five formulations, including Spiriva (tiotropium), Spiolto (tiotropium–olodaterol), Striverdi (olodaterol), Berodual (ipratropium–fenoterol hydrobromide), and Combivent (albuterol–ipratropium) [4]. Only tiotropium was tested in the present study. Aerosol generated with other formulations might be different; thus, our results may not apply to all SMI products. Secondly, due to the lack of commercial options, only one brand of commercial SMI in-line adapter was tested. This bench study evaluated the drug dose with a steady breathing pattern. Clinical trials of drug deposition in the lungs and its correlation to therapeutic effects are needed.

## 5. Conclusions

The drug delivery of the SMI in an intubated lung model was greatly reduced compared with that in a spontaneous breathing model. Among the different methods with the SMI connected to a ventilator system, the in-line adapter yielded the greatest delivered dose, but only with expiratory synchronization. A T-adapter without valve connected to the SMI resulted in an unacceptable leak volume during ventilation and should not be used. Based on low dose efficiency, SMI administration during mechanical ventilation should be avoided until more efficient adapter alternatives are available. Further study on the relationship among delivered drug dose and therapeutic effects and the optimal method of delivery to patients with non-invasive ventilation or tracheostomized patients with spontaneous breathing is desired.
